# ENPP1, an Old Enzyme with New Functions, and Small Molecule Inhibitors—A STING in the Tale of ENPP1

**DOI:** 10.3390/molecules24224192

**Published:** 2019-11-19

**Authors:** Kenneth I. Onyedibe, Modi Wang, Herman O. Sintim

**Affiliations:** 1 Chemistry Department, Institute for Drug Discovery, Purdue University, West Lafayette, IN 47907, USA; konyedib@purdue.edu (K.I.O.); wang3684@purdue.edu (M.W.); 2 Purdue Institute for Inflammation, Immunology, and Infectious Diseases, West Lafayette, IN 47907, USA; 3 Purdue University Center for Cancer Research, West Lafayette, IN 47907, USA

**Keywords:** cyclic dinucleotide, cGAMP, ENPP1, cancer

## Abstract

Ectonucleotide pyrophosphatase/phosphodiesterase I (ENPP1) was identified several decades ago as a type II transmembrane glycoprotein with nucleotide pyrophosphatase and phosphodiesterase enzymatic activities, critical for purinergic signaling. Recently, ENPP1 has emerged as a critical phosphodiesterase that degrades the stimulator of interferon genes (STING) ligand, cyclic GMP–AMP (cGAMP). cGAMP or analogs thereof have emerged as potent immunostimulatory agents, which have potential applications in immunotherapy. This emerging role of ENPP1 has placed this “old” enzyme at the frontier of immunotherapy. This review highlights the roles played by ENPP1, the mechanism of cGAMP hydrolysis by ENPP1, and small molecule inhibitors of ENPP1 with potential applications in diverse disease states, including cancer.

## 1. Introduction

Ectonucleotide pyrophosphatase/phosphodiesterase 1 (ENPP1), also called PC-1, is a type II transmembrane glycoprotein with nucleotide pyrophosphatase and phosphodiesterase enzymatic activities [1]. In 1970, Takahashi et al. showed that PC-1 (or ENPP1) was a 115 kDa and 230 kDa protein under reducing and non-reducing conditions, respectively [2]. ENPP1 is expressed in many tissues [3] and is critical for purinergic signaling, which plays an important role in the regulation of cardiovascular, neurological, immunological, musculoskeletal, hormonal, and hematological functions in mammals [4,5].

Specifically, purinergic signaling is involved in platelet aggregation, muscle contraction, cell proliferation, migration, differentiation, and apoptosis, as well as in regulating hypoxia and ischemia in tissues [4]. Purinergic receptors are divided into two major families, P1 and P2 receptors [6]. P1 receptors are mediated by adenosine, while P2 receptors (P2X and P2Y) are mediated by nucleotides, such as ATP and uridine-5’-triphosphate (UTP) [6]. The extracellular levels of these nucleotides are tightly controlled by their hydrolysis, which is mediated by membrane-bound ENPP1 [7]. ENPP1 catalyzes the hydrolysis of ATP or GTP to AMP or GMP, while generating inorganic pyrophosphates (PPi) [8]. Inorganic pyrophosphates inhibit bone and cartilage mineralization [7,9]. Therefore, the generation of PPi by ENPP1 inadvertently makes ENPP1 a central regulator of bone and cartilage development in mammals [7]. Thus, many inherited mineralization, calcium handling or calcification-related disorders have been linked to loss-of-function mutations in ENPP1, including autosomal recessive hypophosphatemic rickets type 2 (ARHR2), ossification of the posterior longitudinal ligament of the spine, generalized arterial calcification of infancy (GACI), arterial calcification due to CD73 deficiency (ACDC), and pseudoxanthoma elasticum (PXE), also referred to as Gronblad-Strandberg Syndrome (GSS) in some reports [10,11,12,13]. Some classical manifestations of these diseases, such as vascular involvement, are common in ACDC, PXE, and GACI [13,14,15]. These conditions are all ectopic mineralization disorders that occur in the presence of ENPP1 mutations, and have led to the hypothesis that PXE and GACI are actually different manifestations of the same clinical spectrum [13,15]. Recently, Staretz-Chacham et al. reported a fatal multisystemic phenotype of GACI that mimics severe congenital infections and was caused by a novel homozygous ENPP1 mutation [16]. Inactivating mutations of ENPP1, which cause ARHR2, have also been shown to increase the transcription of fibroblast growth factor (FGF) 23 in osteoblasts and osteocytes by a mechanism that has not been fully characterized [10,17,18]. Fibroblast growth factor 23 released from osteoblasts and osteocytes regulates vitamin D metabolism and phosphate homeostasis [17,19]. Additionally, Cole diseases, as well as recently characterized forms of dyschromatoses, have been linked to mutations in ENPP1 [11,20]. Interestingly, Chourabi et al. reported that dyschromatoses patients with ENPP1 mutations also consistently had alterations in their melanocyte development and pigmentation signaling pathways [20]. In addition, insulin resistance, a fundamental starting point for most metabolic diseases, has been linked with ENPP1 mutations in several studies [7,21,22,23]. Huesa et al. observed that ENPP1 knockout mice resisted the expected progression to obesity and insulin resistance despite prolonged high-fat diets [24].

**Figure 1:**
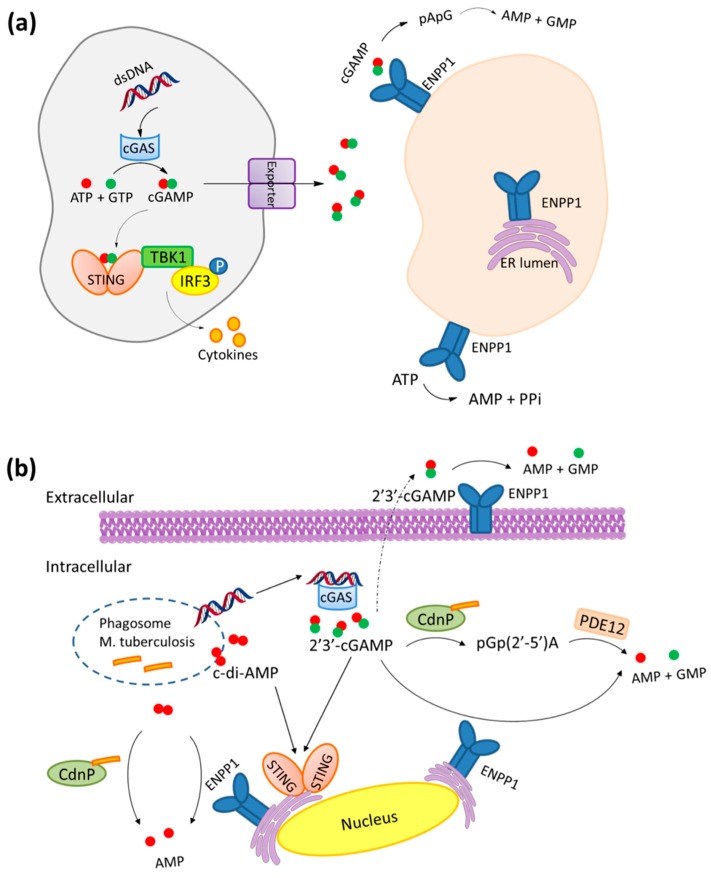
(**a**) Scheme of Ectonucleotide pyrophosphatase/phosphodiesterase 1 (ENPP1) function in the cyclic GMP–AMP synthase (cGAS)–stimulator of interferon genes (STING) pathway. (**b**) Scheme for cyclic dinucleotide signaling and inhibition of cGAS–STING pathway by *M. tuberculosis’* cyclic dinucleotide phosphodiesterase (CdnP). Figure 1b adapted from Reference [24] with permission from Springer Nature Limited, Copyright 2016.

**Figure 2:**
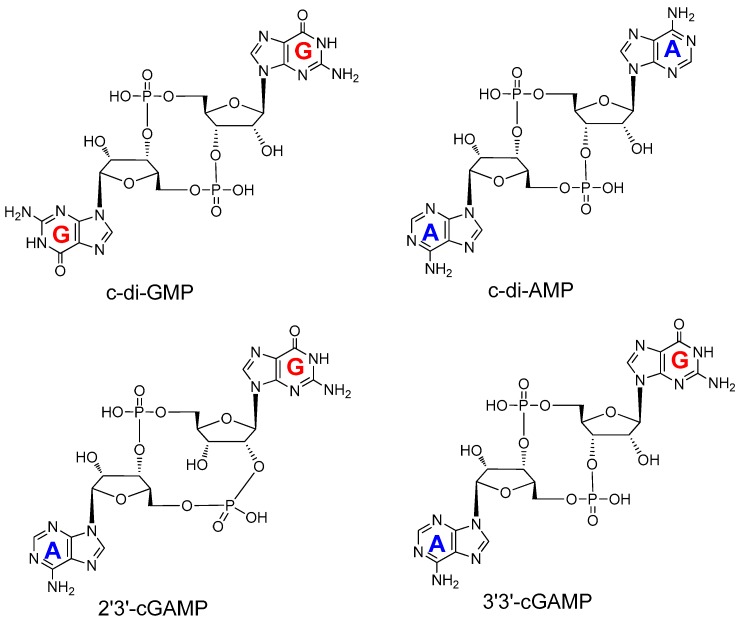
Structures of different cyclic dinucleotides: c-di-GMP, c-di-AMP, 2′3′-cGAMP, and 3′3′-cGAMP.

Recently, ENPP1 has been found to play an important role in the immunological responses to various stimuli through the cyclic GMP–AMP synthase (cGAS)–stimulator of interferon genes (STING) pathway [25]. Damage associated molecular patterns (DAMPs) as well as pathogen associated molecular patterns (PAMPs) activate the immune system via STING [26]. cGAS senses cytosolic DNA and catalyzes the conversion of GTP and ATP to cyclic GMP–AMP (cGAMP) [27,28]. Subsequently, 2′3′-cGAMP activates STING to initiate an inflammatory response via the TANK-binding kinase 1 (TBK1)–Interferon Regulatory Factor (IRF) 3 pathway to produce type 1 interferons (IFNs) and other cytokines [27]. A link between the cGAS–STING pathway and ENPP1 has emerged whereby the hydrolysis of cGAMP by ENPP1 attenuates cGAS–STING signaling [25,29,30,31,32] (Figure 1a). Li et al. reported that the half-life of cGAMP is largely dependent on ENPP1 by demonstrating a much longer cGAMP half-life in ENPP1 knockout mice [32]. Bisphosphothionate analogs of 2’3’-cGAMP, which are resistant to ENPP1 hydrolysis, were shown to activate STING more than 10-fold, compared to 2′3′-cGAMP [32], thus, implying that delayed or reduced cGAMP hydrolysis by inhibition of ENPP1 would significantly increase the activation of STING. Wang et al. recently showed that ENPP1 inhibition attenuates pseudo-rabies infections through alteration of cGAMP homeostasis [30]. Pseudo-rabies is a viral infection, and it has recently emerged that some other viruses, such as pox virus, possess cytosolic immune nucleases (poxins), which degrade 2’3’-cGAMP and make it unable to activate STING [33]. These poxins are believed to be viral self-protective enzymes that prevent 2′3′-cGAMP from activating STING [33]. Likewise, Dey et al. reported that *Mycobacterium tuberculosis* (Mtb) could inhibit STING activation and evade host immunity via the concerted action of cyclic dinucleotide phosphodiesterase (CdnP) and ENPP1 (Figure 1b) [31]. Cyclic-di-AMP and cyclic-di-GMP (Figure 2) from invading pathogens also activate the cGAS–STING pathway in the same manner as 2′3′-cGAMP [27].

In addition to being a target for bone [7,34], cardiovascular [35], and metabolic diseases [23], ENPP1 has now emerged as a therapeutic target for cancers, as discussed below [36]. ENPP1 plays a regulatory function in immune cells such as neutrophils, macrophages, dendritic cells, natural killer cells, and B lymphocytes [37,38]. ENPP1 expression is heightened in M2 macrophages in the presence of cancer and promotes tumor growth and spread [30,39]. The location of the gene for ENPP1 is the 6q22-q23 position, which is a region that has been found to be amplified in many tumors, including breast and neural brain cancers [40,41]. Clearly, ENPP1 inhibitors would play an important role in cancer immunotherapy. Although other excellent reviews on ENPP1 inhibitors have been published, these were published before the new role of ENPP1 in modulating the immunomodulatory ligand, cGAMP, was discovered or fully elucidated [42]. Here, we present the current state of knowledge of ENPP1 and inhibitors, which could be used to modulate ENPP1 function. 

## 2. Mechanism of Hydrolysis of ATP and 2′3′-cGAMP by ENPP1

**Figure 3:**
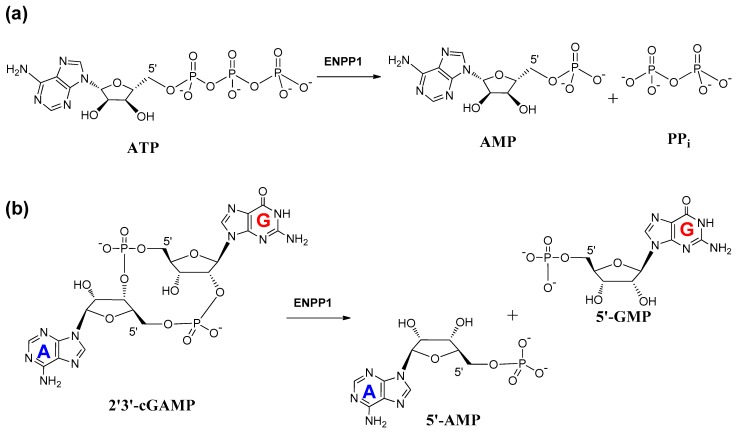
Hydrolysis of substrates (**a**) ATP and (**b**) 2′3′-cGAMP by ENPP1.

ENPP1 is located on plasma membranes and ER lumen [43]. One of the main substrates of ENPP1 is ATP, a pro-inflammatory signaling molecule. The level of ATP is relatively high in the tumor microenvironment due to the presence of damaged or dying cells [44]. Hydrolysis of ATP by ENPP1 would produce AMP and PPi extracellularly (Figure 3a) to prevent bone mineralization [45,46]. AMP is then dephosphorylated to adenosine by the ecto-5′-nucleotidase (CD73) in the canonical adenosinergic pathway [47].

**Figure 4:**
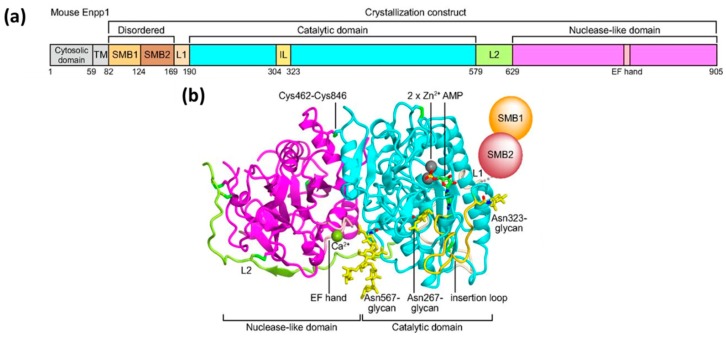
(**a**) Domain organization of mouse ENPP1. (**b**) Crystal structure of the extracellular domain of ENPP1 in complex with AMP. Catalytic domain, cyan; nuclease-like domain, magenta; L1, wheat; L2, yellow-green; EF hand-like motif, pink; insertion loop, gold. AMP and *N*-glycans are shown as green and yellow sticks, respectively. The bound zinc and calcium ions are shown as gray and yellow-green spheres, respectively. Disulfide linkages are shown as sticks. The two somatomedin B (SMB)-like domains are shown by circles, as they are disordered in the crystal structure [21]. Reproduced from Reference [21] with permission from National Academy of Sciences, Copyright 2012.

Kato et al. reported the crystal structures of the ENPP1 extracellular domain bound to four nucleotide monophosphates (MP), (adenosine (AMP), thymidine (TMP), guanosine (GMP), and cytidine (CMP)) at resolutions of 2.7–3.2 Å, in 2012 [21]. The ENPP1 structure has two N-terminal somatomedin B (SMB)-like domains (SMB1 and SMB2), two linker regions (L1 and L2), a catalytic domain, and a nuclease-like domain (Figure 4a). There are three glycosylation sites for ENPP1, which reinforce the domain interaction [21]. It was believed that the SMB-like domains functioned as a transmembrane anchor and were not involved in the enzymatic activity of ENPP1. On the other hand, ENPP1 is a Ca^2+^- and Zn^2+^-dependent enzyme, and enzymatic activity is strongly correlated to the concentration of calcium and zinc ions [21]. The nuclease-like domain of ENPP1 contains a calcium ion-binding EF hand motif, which is found in a large family of calcium-binding proteins. The calcium ion is chelated by Asp780, Asp782, Asp784, and Asp788, and the carbonyl group of Arg786 to form an EF hand-like motif (Figure 4b). The catalytic domain chelates two zinc ions in the active site, a location which plays an important role in the function of ENPP1 [21]. The phosphate group of ATP binds in between the two zinc ions to trigger the bond cleavage process and produce AMP and PPi. The nitrogen-6 atom of AMP interacts with Trp304 and Asp308 by a H_2_O-mediated hydrogen bond network. However, no hydrogen-bonding network is formed for TMP, GMP, and CMP, thereby resulting in the selectivity of ENPP1 for ATP. Hence, ATP is the most efficient and well-investigated substrate for ENPP1. There are other reported natural substrates of ENPP1 including UTP, diadenosine tetraphosphate (AP_4_A), cyclic adenosine monophosphate (cAMP), and 2′3′-cGAMP, but not 3′3′-cGAMP (Figure 2) [48]. It is already known that cyclic nucleotides, such as cAMP and cGMP, are exported by multidrug resistance proteins (MRPs) [49,50,51]. Recently, it was also shown that some cyclic dinucleotides (such as cyclic-di-AMP) were exported by MRPs [52]. Cyclic nucleotides are degraded by phosphodiesterases (PDEs) 1 to 11 [53]. However, these PDEs do not degrade cyclic dinucleotides, such as 2′3′-cGAMP. Viral poxins and ENPP1 are now the known hydrolytic enzymes of 2′3′-cGAMP [32,33]. ENPP1 hydrolyzes 2′3′-cGAMP with a kinetic rate constant that is similar to the hydrolysis of ATP (Figure 3b) [32].

**Figure 5:**
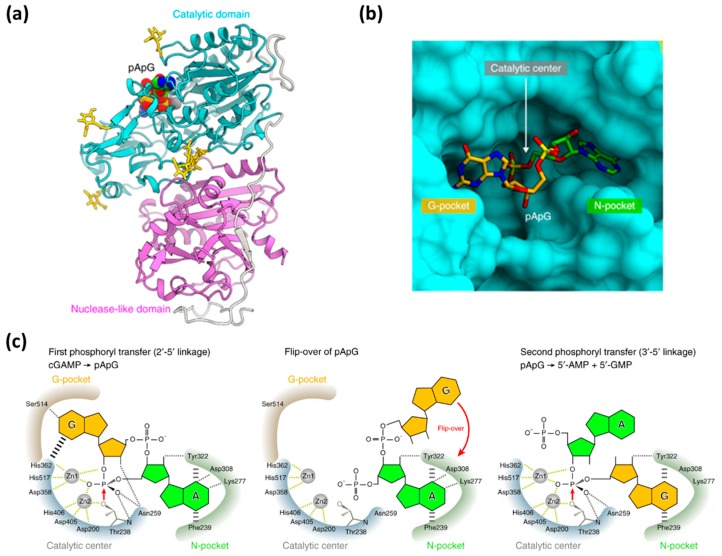
(**a**) Overall structure of ENPP1 in complex with phosphoadenylyl guanosine (pApG). The N-linked sugars are shown as yellow sticks. (**b**) Binding of pApG to the ENPP1 active site. (**c**) Proposed mechanism of the ENPP1-catalyzed 2′3′-cGAMP degradation [25]. Reproduced from Reference [25] with permission from Springer Nature Limited, Copyright 2018.

**Figure 6:**
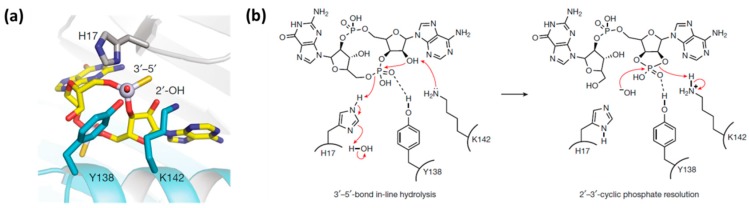
(**a**) Crystal structure of Vaccinia virus poxin–2′,3′-cGAMP complex highlighting residues involved in 3′-5′-bond hydrolysis. (**b**) Mechanism of poxin metal-independent hydrolysis [33]. Reproduced from Reference [33] with permission from Springer Nature Limited, Copyright 2019.

To understand the mechanism of 2′3′-cGAMP hydrolysis by ENPP1, Kato et al. investigated the crystal structure of ENPP1 in complex with 2′3′-cGAMP (Figure 5) [25]. ENPP1 utilizes two Zn^2+^ ions, coordinated by Asp358/His362/His517 and Asp200/Asp405/His406, to interact with the phosphate oxygen. The enzyme hydrolyzes the 2′-5′ phosphodiester bond of 2′3′-cGAMP first to form phosphoadenylyl guanosine (pApG) and then a second hydrolysis produces 5′-AMP and 5′-GMP [25]. Recently, Eaglesham et al. described cGAMP hydrolysis by viral and metazoan poxins [33]. However, the mechanism for cGAMP hydrolysis by poxin is different from that of ENPP1. Firstly, cGAMP binds to ENPP1 as the anion and one of the phosphates coordinates to two zinc ions ligated by histidine and aspartate residues, as shown in Figure 5c, whereas, cGAMP binds to poxin as the acid with no active site metal (Figure 6a) [33]. Additionally, for ENPP1, an Oγ atom of a threonine residue (Thr 238) is the nucleophile for hydrolysis, whereas in the poxin mechanism, the nucleophile for phosphate cleavage is the 2’OH of the cGAMP. In addition, for the poxin hydrolysis mechanism, histidine (H17) and tyrosine (Y138) act as general acids, whereas lysine (K142) acts as a general base to deprotonate 2’OH on cGAMP. The first stage of the poxin hydrolysis leads to a 2′-3′-cyclic phosphate, which is then hydrolyzed by an active site hydroxyl ion (OH^−^), as seen in Figure 6b. Another difference between the poxin and ENPP1 mechanisms of hydrolysis of cGAMP is that ENPP1 cleaves the 2′-5′ phosphodiester bond first, followed by cleavage of the 3′-5′ bonds, whereas poxins only cleave the 3′-5′ phosphodiester bond [25,33]. Notwithstanding these differences, both types of hydrolysis ensure that the hydrolyzed 2′3′-cGAMP is not able to activate the STING pathway.

## 3. STING and ENPP1 in Cancer

**Figure 7:**
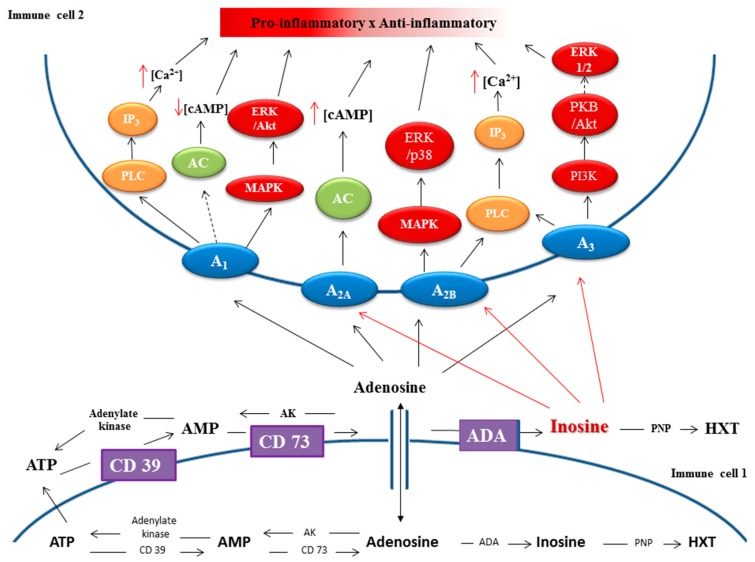
Scheme of the purinergic system in inflammation. Intracellular adenosine is produced from AMP or S-adenosylhomocysteine, which is then exported to the extracellular environment by nucleoside transporters, to stimulate several adenosine receptor-mediated signaling pathways. Extracellular adenosine acts as the signaling molecule that binds and stimulates the cell surface adenosine receptors (A_1_, A_2A_, A_2B_, and A_3_) and triggers both pro-inflammatory and anti-inflammatory responses. A_1_, adenosine A1 receptor; A_2A_, adenosine A2A receptor; A_2B_, adenosine A2B receptor; A_3_, adenosine A3 receptor; AC, adenylate cyclase; cAMP, cyclic adenosine monophosphate; ERK1/2, extracellular signal-regulated kinases 1 and 2; IP3, inositol triphosphate; MAPK, mitogen-activated protein kinase; PKB, protein kinase B; PLC, phospholipase C; PI3K, Phosphatidylinositide 3-kinase; ATP, adenosine triphosphate; AMP, adenosine monophosphate; HXT, hypoxanthine; AK, adenosine kinase; ADA, adenosine deaminase; PNP, purine nucleoside phosphorylase; ENTs, equilibrate nucleoside transporters; CD39, ectonucleoside triphosphate diphosphohydrolase; Inhibition →; Activation - - →[70]. Reproduced from Reference [70] with permission from Intech Open, Copyright 2014.

Several studies have shown that STING pathway activation could stimulate anti-inflammatory T cell responses via the induction of indoleamine 2,3 dioxygenase (IDO), which could in turn cause immune suppression in the tumor microenvironment [54,55,56,57,58]. Indoleamine 2,3 dioxygenase 1 (IDO1) is an example of an IFN-stimulated gene (ISG) responsive to IFNs via IFN-response elements found in the mammalian IDO1 gene promoters. IDO is a tryptophan-catabolizing enzyme, which promotes the activation of CD4^+^ regulatory T cells mainly of the Foxp3-lineage and the subsequent suppression of effector and helper T cell functions [56]. In dendritic cells, IDO enhances immune tolerance by induction of transforming growth factor beta (TGF β) [55,57,59]. In a Lewis lung carcinoma mice model, Lemos et al. showed that knockout of STING caused enhanced killing of cancer cells due to increased CD8^+^ T cell activity, reduced myeloid suppressor cell infiltration, and high levels of IL10 production in the tumor microenvironment [55]. However, the induction of IDO did not produce the same effect in STING-deficient EL4 thymoma, B16 melanoma, and in neo-antigen-expressing lung carcinoma [55]. The immune-suppressive effect of IDO that leads to tumorigenesis is promoted in tumors with low antigenicity [55]. Hence, when tumor antigenicity is low, STING activation induces immune-regulatory responses via IDO predominantly, whereas, in tumors with high antigenicity, immune-stimulatory responses are enhanced [55]. Similarly, and perhaps, more importantly, adenosine produced by the adenosinergic pathway exhibits significant immunosuppressive effects in the tumor microenvironment and contributes to tumor progression [60,61]. ATP is rapidly dephosphorylated in a stepwise manner in the extracellular milieu by the ecto-nucleotidases, CD39 and CD73 [53]. Typically, CD39 converts ATP to ADP and ADP to AMP, while CD73 dephosphorylates AMP to adenosine (Figure 7) [60,62]. Additionally, in the usually hypoxic tumor microenvironment, hypoxia induces further CD39- and CD73-mediated adenosine production [63,64]. To add to this, hypoxia also inhibits breakdown of adenosine and potentiates adenosine release by downregulating adenosine kinase [61,65]. The increased production of adenosine and the inhibition of its breakdown ultimately leads to much higher levels of adenosine in tumors when compared to normal tissues [66]. The excess adenosine produced by these mechanisms essentially turns off both the innate and adaptive immune responses via G-protein-coupled A_2A_ and A_2B_ adenosine receptors that stimulate cyclic AMP, consequently leading to decreased production of proinflammatory cytokines and increased synthesis of anti-inflammatory cytokines [44,65]. In cervical cancer-derived mesenchymal stromal cells (MSC), de Lourdes Mora-Garcia et al. demonstrated that cytotoxic T lymphocyte effector activity, including proliferation and production of IFN-γ+, were inhibited by adenosine in a dose-dependent manner [65]. Other studies by Garcia-Rocha et al. also reported that MSC derived from cervical cancer tumors induced the expression and secretion of anti-inflammatory cytokines, such as TGF-β1 and IL-10, in cervical cancer cells, thereby protecting the cells from T cell cytotoxicity [67]. The hydrolysis of cGAMP by ENPP1 leads to the production of AMP, which eventually contributes to a more profound immunosuppression via the subsequent dephosphorylation of AMP to adenosine by CD73 [47]. The role of ENPP1 in cancer is exemplified by the observations of enhanced tumor metastasis to the bone from breast cancer, for example, by over-expression of ENPP1 [41]. The significance of ENPP1 and CD73-mediated production of adenosine is further demonstrated by several reports of resistance to carcinogenesis or metastasis by mice deficient in either CD73 or ENPP1 [41,63,68,69].

## 4. Inhibitors of ENPP1

### 4.1. Nucleotide-Based Inhibitors of ENPP1

**Figure 8:**
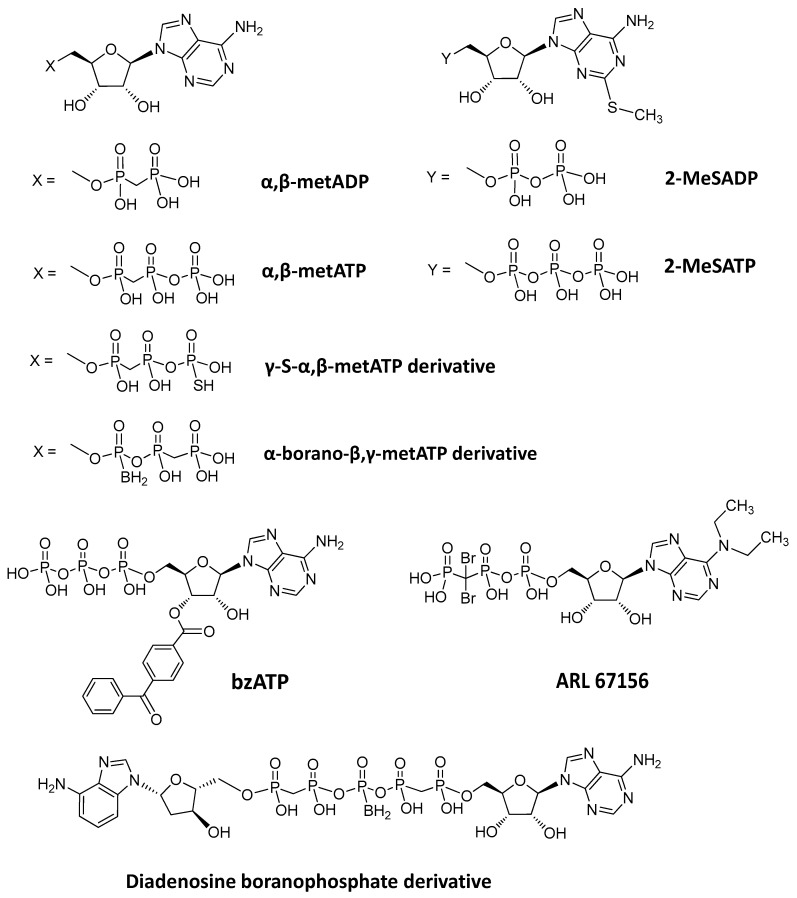
Structures of nucleotide-based ENPP1 inhibitors.

The therapeutic potential of ENPP1 inhibitors has increased with the discovery of ENPP1’s role in modulating the cGAS–STING pathway. In the past years, a few nucleotide-based ENPP1 inhibitors have been developed, which are mostly substrate analogs (Figure 8), such as adenine nucleotide derivatives [71,72,73,74,75]. These adenine nucleotide analog inhibitors of ENPP1 generally exhibit a competitive type of inhibition as their structures are similar to natural ENPP1 substrates [42]. Additionally, the inhibition properties of these nucleotide analogues appear to be similar. Using human soluble ENPP1 and ATP as substrates, K_i_ for compounds α,β-metADP, α,β-metATP, 2-MeSADP, and 2-MeSATP, bzATP ranged from 13 to 32 µM, and was considered to be moderately potent [42,71,72]. Other nucleotide-based inhibitors have also been reported, such as γ-S-α, β-metATP derivatives, ARL 67156, α-borano-β, γ-metATP derivatives, and diadenosine boranophosphate derivatives. These other nucleotide inhibitors used p-Nph-5′-TMP or p-Nph-5′-AMP as substrates to achieve colorimetric detection of activity [73,74,75].

However, the high acidity precludes oral bioavailability and limits the applicability of nucleotide-based inhibitors. Furthermore, the NPP1 selectivity of these inhibitors against other ectonucleotidases is not well understood. Since the structure of nucleotide-based inhibitors is similar to the structure of natural substrates, the possibility of off-target biological effects, such as P2 purinergic receptor activation, could also be enhanced. Consequently, nucleotide-based inhibitors may not be ideal lead candidates for the development of translatable ENPP1 inhibitors.

### 4.2. Non-Nucleotide-Based Inhibitors of ENPP1

Many of the reported nucleotide-based inhibitors become negatively charged at physiological pH and have very poor oral bioavailability [76]. Nucleotide-based inhibitors also have very challenging synthesis and purification steps [76]. Non-nucleotide ENPP1 inhibitors have also been reported (Table 1). For example, polyoxometalates [TiW11CoO40]^8−^ were found to be one of the most potent ENPP1 inhibitors, with a K_i_ of 1.46 nM (0.00146 µM), when compared to other non-nucleotide human soluble enzyme inhibitors, such as reactive blue 2 (RB2), quinazoline derivative, and suramin, with a K_i_ of 0.141, 0.215, and 0.780 µM, respectively [42]. Among the human membrane-bound enzyme inhibitors with ATP as a substrate, suramin had a superior K_i_ of 0.26 µM relative to RB2, with a K_i_ of 0.52 µM. In contrast to nucleotide-based inhibitors, which exhibit competitive inhibition, suramin showed uncompetitive inhibition against the human soluble enzyme [42]. However, heparin, one of the first ENPP1 inhibitors, described about 2 decades ago, was reported to have a relatively high IC_50_ of 100 µM [77]. Several other non-nucleotide-based inhibitors have been reported, where other substrates, such as ATP with a radioactive phosphorus atom at γ-position of the triphosphate ([γ^−32^P]ATP), etheno-diadenosine diphosphate, bis(*p*-nitrophenyl) phosphate, *p*-nitrophenyl phenyl phosphate, *p*-Nph-5′-TMP, or *p*-Nph-5′-AMP were used (Table 1) [42]. The K_i_ values in these reports varied widely and ranged from 0.0593 to 1400 µM (see Table 1) [71,76,78,79,80,81].

Most recently, in 2019, two new sulfamate derivatives were reported by Semreen et al. and El-Gamal et al., with an IC_50_ of 0.387 and 0.29 µM, respectively [82,83]. The sulfamate and sulfonate derivate described by El-Gamal was synthesized from a backbone of raloxifene hydrochloride [83]. Raloxifene is an FDA-approved selective estrogen receptor modulator used in the prevention and treatment of postmenopausal osteoporosis, and is known to reduce the risk of breast cancer [84]. Earlier in 2018, Forcellini et al. reported several novel quinazoline-4-piperidine sulfamide analogs as inhibitors of ENPP1 [85]. Among these, meta-pyridine substituted compound 7c was the most potent compound, with a K_i_ of 58 nM (0.058 µM) [85]. More recently, Weston et al. reported a new ENPP1 inhibitor, SR-8314, which promotes STING activation [86]. The K_i_ value of SR-8314 against ENPP1 activity was reported as 0.079 µM. It was also shown that SR-8314 has anti-tumor activity with more CD3^+^, CD4^+^, and CD8^+^ T cells found in SR-8314 treatments compared to controls [86]. Baird et al. developed another selective ENPP1 inhibitor, MV-626, which prevents the hydrolysis of cGAMP, and increases STING activation [87]. Therapeutic doses of MV-626 did not show toxicity in mice [87]. In combination with radiation therapy, MV-626 increased overall survival, and the majority of test animals had durable tumor cures [87]. Currently, the ideal doses, intensities, and durations of such therapies has not been established. Generally, hyperactivation of host immune responses and ectopic calcifications by ENPP1 inhibition might be a concern, as discussed in a previous review [36]. However, ENPP1 knockout mice have remained viable, lending credence to the potential use of ENPP1 inhibitors without debilitating adverse outcomes. The addition of these new, more potent inhibitors into the growing array of ENPP1 inhibitors, is a reflection of the therapeutic usefulness of ENPP1 inhibition in immunotherapy.

## 5. Conclusions

In this review, we have discussed the mechanism and hydrolysis of the relatively new ENPP1 substrate, cGAMP, and that of ATP. We have also highlighted the typical and recently reported ENPP1 inhibitors, based on their classification as either nucleotide-based or non-nucleotide-based inhibitors. Since STING activation is a promising therapeutic strategy to cure cancer, more and more compounds that activate the STING pathway have been reported. ENPP1, as a highly potent cGAMP-degradation enzyme, makes the application of ENPP1 inhibitors for anti-tumor therapy a very topical issue. For virally-infected hosts, small molecule inhibitors of ENPP1 would also need to inhibit viral poxins for maximum efficacy.

## Figures and Tables

**Table 1:** Non-nucleotide-based inhibitors ^‡^.

Inhibitor (I)	Structure	Substrate	Enzyme	K_i_/ IC_50_^a^	Inhibition	Ref
				(µM)	Type	
**Polysaccharides**	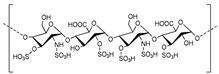					
**Heparin**		ATP	Human, soluble	100^a^	Competitive	[77]
		*p*-Nph-5′-TMP	Human, soluble	1.0^a^	Competitive	[77]
		εAP2A	Unspecified rat NPPs	25	Competitive	[88]
**Polysulfonates**						
Suramin	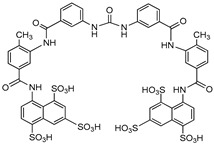	P^32^-ATP	Unspecified rat NPPs	61–83^a^	Not defined	[79]
		ATP	Human, membrane-bound	0.26	Not defined	[89]
		*p*-Nph-5′-TMP	Human, membrane-bound	8.67^a^	Not defined	[90]
		*p*-Nph-5′-TMP	Human, soluble	1.07	Uncompetitive	[71]
		*p*-Nph-5′-AMP	Human, soluble	1.03	Uncompetitive	[71]
		ATP	Human, soluble	0.78	Uncompetitive	[71]
Reactive blue 2	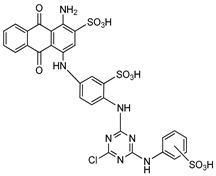	P^32^-ATP	Unspecified rat NPPs	9–15^a^	Not defined	[79]
		ATP	Human, membrane-bound	0.52	Not defined	[89]
		ATP	Human, soluble	0.14	Noncompetitive	[71]
		*p*-Nph-5′-TMP	Human, soluble	0.198	Noncompetitive	[71]
		*p*-Nph-5′-AMP	Human, soluble	0.176	Noncompetitive	[71]
**Polyoxometalates**						
[TiW11CoO40]8^−^		ATP	Human, soluble	0.00146	Noncompetitive	[91]
**Other Heterocyclic compounds**						
PPADS	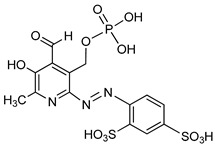	P^32^-ATP	Unspecified rat NPPs	9–15^a^	Not defined	[79]
Biscoumarin derivative	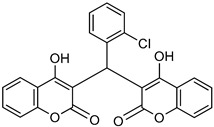	*p*-NPPP	Human, soluble	50–1000	Noncompetitive	[92]
		bis(*p*-NP)P	Snake venom	8–1150	Noncompetitive	[92]
Oxadiazole derivatives	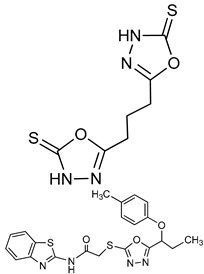	*p*-NPPP	Human, soluble	150–850	Noncompetitive	[93]
		bis(*p*-NP)P	Snake venom	100–1400	Noncompetitive	[93]
		*p*-Nph-5′-TMP	Human, membrane-bound	1.9–5.5^a^	Noncompetitive	[94]
Quinazoline derivative	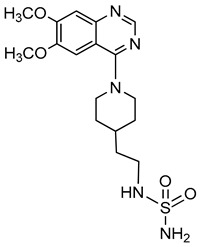	ATP	Not determined	0.036–5.98^a^	Not defined	[95]
		*p*-Nph-5′-TMP	Human, membrane-bound	0.059–0.110	Noncompetitive	[80]
		*p*-Nph-5′-TMP	Human, soluble	0.06^b^	Competitive	[71]
		*p*-Nph-5′-AMP	Human, soluble	0.42^b^	Competitive	[71]
		ATP	Human, soluble	0.215	Competitive	[71]
Triazole derivative	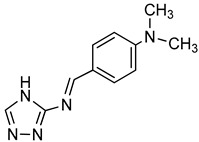	bis(*p*-NP)P	Snake venom	132–1164^a^	Not defined	[96]
Thioacetamide derivative	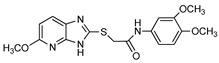	ATP	Human, soluble	5.34–89.7	Competitive	[76]
		*p*-Nph-5′-TMP	Human, soluble	0.005–11.0	Competitive	[76]
Isoquinoline derivative	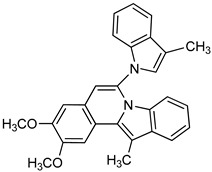	*p*-Nph-5′-AMP	Human, soluble	14.9	Competitive	[71]
Thiadiazolopyrimidinone derivative	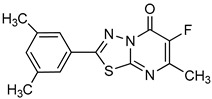	*p*-Nph-5′-TMP	Human, membrane-bound	0.36–2.81^a^	Competitive	[81]
		*p*-Nph-5′-TMP	Human, membrane-bound	0.31–2.26^a^	Competitive	[90]
Thiazolobenzimidazolon-e derivative	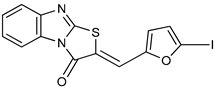	ATP	Human, soluble	0.467–0.981	Uncompetitive	[78]
Sulfamate derivatives	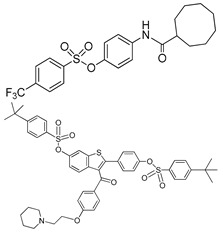	*p*-Nph-5′-TMP	Human, soluble	0.29^a^		[83]
		*p*-Nph-5′-TMP		0.387^a^	Competitive	[82]
SR 8314	Not disclosed	ATP	Human, soluble	0.079	Not determined	[86]
MV 626	Not disclosed	ATP		5–18		[87]

^‡^ Adapted from Lee et al. 2017 [42] (updated with more recent inhibitors) with permission from the Royal Society of Chemistry, Copyright 2017; ^a^ IC50 values, ^b^ Value determined with the most potent derivative of quinazolines.
